# Deciphering the Fine-Tuning of the Retinoic Acid-Inducible Gene-I Pathway in Teleost Fish and Beyond

**DOI:** 10.3389/fimmu.2021.679242

**Published:** 2021-04-28

**Authors:** Raphaël Jami, Emilie Mérour, Annie Lamoureux, Julie Bernard, Jean K. Millet, Stéphane Biacchesi

**Affiliations:** University Paris-Saclay, INRAE, UVSQ, VIM, Jouy-en-Josas, France

**Keywords:** interferon, RIG-I-like receptors (RLRs), innate response, immune homeostasis, fish

## Abstract

Interferons are the first lines of defense against viral pathogen invasion during the early stages of infection. Their synthesis is tightly regulated to prevent excessive immune responses and possible deleterious effects on the host organism itself. The RIG-I-like receptor signaling cascade is one of the major pathways leading to the production of interferons. This pathway amplifies danger signals and mounts an appropriate innate response but also needs to be finely regulated to allow a rapid return to immune homeostasis. Recent advances have characterized different cellular factors involved in the control of the RIG-I pathway. This has been most extensively studied in mammalian species; however, some inconsistencies remain to be resolved. The IFN system is remarkably well conserved in vertebrates and teleost fish possess all functional orthologs of mammalian RIG-I-like receptors as well as most downstream signaling molecules. Orthologs of almost all mammalian regulatory components described to date exist in teleost fish, such as the widely used zebrafish, making fish attractive and powerful models to study in detail the regulation and evolution of the RIG-I pathway.

## Introduction

The antiviral innate immune response in vertebrates is mediated by type I interferon (IFN) and its actions as an autocrine signal for the infected cell and as a paracrine “early warning” signal to neighboring cells (1, 2). This host response against virus infection is characterized by the induction of a rapid non-specific antiviral state that blocks virus replication and spread. The IFN system is remarkably well conserved in vertebrates which highlights its critical importance (3). Teleost fish possess functional orthologs of pattern-recognition receptors (PRRs). Toll-like receptors (TLRs) and C-type lectin receptors (CLRs) detect pathogens in the extracellular or the endosomal compartments, while retinoic acid-inducible gene-I (RIG-I)-like receptors (RLRs), Nod-like receptors (NLRs), and cytoplasmic DNA sensors serve as intracellular PRRs. These sensors are able to detect distinct viral molecular patterns, such as nucleic acids or viral proteins, collectively known as pathogen-associated molecular patterns (PAMPs). They synergistically trigger the activation of multiple signaling cascades that induce the production of IFN and other cytokines, thereby establishing an antiviral state and shaping an appropriate adaptive immune response. Among the PRRs, RLRs play a key role in sensing viral RNA in the cytosol and are essential in the early induction of IFN (4, 5). The ability of IFNs to restrict virus replication in mammals is largely mediated through the induction of hundreds of interferon-stimulated genes (ISGs), collectively referred as the “interferome” (6). Similarly, up-regulation of ISGs by IFNs in lower vertebrates has been extensively reported. Several studies point to the maintenance of a stable set of core ISGs during evolution (7) and their key functions for fish defense against viruses (3, 8).

## RIG-I-Like Receptors: From RNA Sensing to IFN Induction

**Figure 1:**
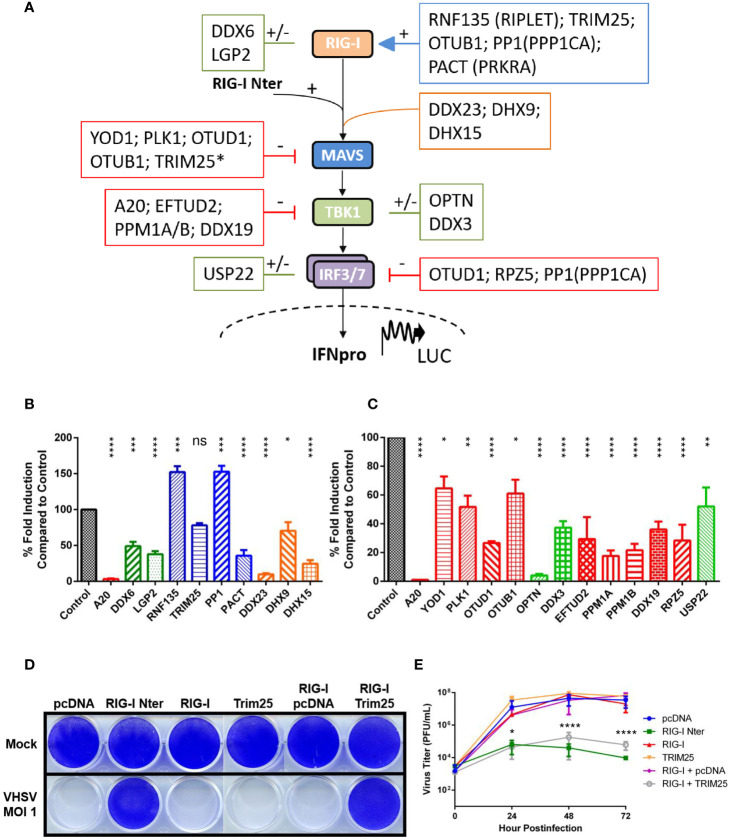
Regulation of RIG-I-mediated signal transduction by a conserved set of cellular proteins in vertebrates. **(A)** Schematic representation of RIG-I pathway including the main downstream components for signal transduction leading to promoter activation and expression of type-I interferon. A set of cellular regulators that are evolutionarily conserved between fish and mammalian species are placed next to their targets. The effect of each regulator is symbolized by (–) for inhibition (in red), by (+) for activation (in blue), or by (+/-) for ambivalent (in green) reported functions on the RIG-I pathway, based on the literature (mainly from studies with mammalian orthologs; see **Table 1**). Other cytosolic sensors or co-receptors involved in the RIG-I pathway are boxed in orange. *Although TRIM25 promotes the degradation of MAVS, this step is required for IRF3 phosphorylation by TBK1 (23). **(B, C)** Fathead minnow orthologs of mammalian regulators were amplified from total RNA extracted from EPC cells, cloned into the eukaryotic expression vectors pcDNA1.1/Amp (Invitrogen) and fully sequenced. Nucleotide sequences of each regulator were deposited in GenBank (see **Table 1** for accession numbers). To test their effect on the RIG-I pathway, EPC cells were transfected with the indicated plasmids (1 μg) together with a luciferase reporter construct driven by the promoter of IFN1 derived from EPC cells (1 μg) and the RIG-I Nter-eGFP inducer and internal transfection control construct 0.5 µg in **(B)** or 1 μg in **(C)**, as previously described (24). Twenty-four hours after transfection, the cells were lysed for luciferase assays. Luciferase activity was measured and normalized to eGFP fluorescence. No significant variation in eGFP expression was observed between each condition. The percentage of fold-induction were calculated as the ratio of stimulated (+ RIG-I Nter) versus unstimulated (− RIG-I Nter) conditions and compared to the induction control (RIG-I Nter + empty vector). Means of at least three independent experiments are shown together with the standard errors. The color coding used for the histograms is the same as the one used in panel **(A)** For statistical analysis, a comparison between groups was performed with a one-way ANOVA and Tukey’s multiple comparison tests using GraphPad Prism (GraphPad, San Diego, CA). Groups that are not significantly different from each other are denoted ns (P > 0.05), whereas those that are significantly different are denoted *(P < 0.05), **(P < 0.01), ***(P < 0.001) or ****(P < 0.0001). **(D, E)** EPC cells were transfected with the indicated plasmids (2 μg each) or an empty vector (pcDNA1.1/Amp) as a control, as previously described (25). All transfection mixtures were adjusted with an empty vector to contain an equal amount of plasmid DNA. Twenty-four hours after transfection, cells were infected with a fish novirhabdovirus, viral hemorrhagic septicemia virus (VHSV) at an MOI of 1 and incubated at 15°C. Cell monolayers were stained with crystal violet 3 days postinfection **(D)**. The culture supernatants from infected cells were collected at different times postinfection and the viral titer was determined by plaque assay **(E)**. Each time point is represented by two independent experiments, and each virus titration was performed in duplicate. Average values are shown. The standard errors were calculated and the error bars are shown. Asterisks indicate significant difference (*p < 0.05; ****p < 0.0001) as determined by two-way ANOVA and Tukey’s multiple comparison tests. ns, not significant.

The sensing of non-self-cytosolic RNA is mediated by RLRs which include RIG-I (DDX58) (9), melanoma differentiation-associated gene 5 (MDA5/IFIH1) (10–12), and laboratory of genetics and physiology 2 (LGP2/DHX58) (13, 14). Notably, RIG-I detects viral replication not only in the cytoplasm, but also in the nuclear compartment (15). In mammals, it is now recognized that most if not all viral infections from RNA and DNA viruses can be recognized by RLRs. RIG-I and MDA5 are DExD/H box RNA helicases comprising three domains; two N-terminal caspase recruitment domains (CARDs) in tandem involved in signal transduction, a central helicase domain and a C-terminal domain (CTD) critical for RNA recognition and autoinhibition of CARDs (16). LGP2 contains a helicase domain but lacks CARDs and thus a signal-transducing activity. LGP2 is a regulator with distinct effects on RIG-I and MDA5. While LGP2 clearly upregulates the signaling activity of MDA5, its action on RIG-I-mediated antiviral signaling remains unclear (13, 17, 18). In fact, LGP2 deficiency has different effects depending on the nature of the viral infection (19, 20). Nevertheless, LGP2 can associate with the C-terminus of TNF receptor associated factors (TRAFs) and can regulate TRAF activity downstream of RIG-I and MDA5, indicating that LGP2 can suppress both MDA5‐dependent and RIG‐I‐dependent signal transduction (21). RLRs are remarkably well conserved in vertebrates and teleost fish possess functional orthologs of human RLRs, including RIG-I, MDA5, and LGP2 (4, 22) as well as several downstream molecules (**Figure 1A** and **Table 1**). Although identified in many fish species belonging to Cypriniformes (e.g. carp and zebrafish), Siluriformes (e.g. channel catfish) and Salmoniformes (e.g. salmon and trout), RIG-I has not been reported in certain fish of the superclass Acanthopterygii (e.g. medaka, tetraodon, pufferfish, stickleback, sea bream and sea bass). It is still unclear whether the RIG-I gene has been lost in some fish species as it has been reported for chicken (105) and Chinese tree shrew (106).

**Table 1:** *Pimephales promelas* RIG-I pathway components and orthologs.

RIG-I Pathway Components of *Pimephales promelas*						
Full Name	Symbol(Synonym)	Target/Partner	GenBank #	*Homo sapiens* GeneID	*Danio rerio* GeneID	SelectedReferences
DExD/H-box helicase 58	DDX58 (RIG-I)	MAVS	FN394062	23586	100333797	(9, 25, 26)
DDX58 CARD domains	RIG-I Nter	MAVS	FN178456			
Interferon induced with helicase C domain 1	IFIH1 (MDA5)	MAVS	MG799354	64135	565759	(10–12, 27, 28)
Mitochondrial antiviral signaling protein	MAVS (IPS1, CARDIF, VISA)	TRAF3 TBK1	FN178455	57506	562867	(25, 29–33)
TANK binding kinase 1	TBK1	IRF3/7	LT174673	29110	692289	(24, 34–38)
Interferon regulatory factor 3	IRF3	IFN promoter	**MN781134**	3661	564854	(39–44)
Interferon regulatory factor 7	IRF7	IFN promoter	**MN781135**	3665	393650	(34, 45–47)
Interferon 1 promoter region	IFN1 promoter	_	HE856618	IFNα/β promoter	DQ855952^1^	(48–50)
Interferon 1	IFN1	IFN receptor	FN178457	IFNα/β	360134	(2, 25, 51, 52)
**RIG-I Pathway Regulators/Co-receptors/Receptors of *Pimephales promelas***						
DExH-box helicase 58	DHX58 (LGP2)	RIG-I	**MW591879**	79132	100148871	(13, 17, 19, 21, 27, 49, 53–55)
DEAD-box helicase 6	DDX6	RIG-I	**MW591868**	1656	564633	(56, 57)
Ring finger protein 135	RNF135 (RIPLET)	RIG-I	**MW591864**	84282	101882927	(58–63)
Tripartite motif containing 25	TRIM25	RIG-I	LT174676	7706	393144	(23, 64–66)
OTU deubiquitinase, ubiquitin aldehyde binding 1	OTUB1	RIG-I TRAF3	**MW591878**	55611	436684	(67–69)
Protein phosphatase 1 catalytic subunit alpha	PPP1CA (PP1A)	RIG-I IRF3/7	**MW591866**	5499	407980	(70, 71)
Protein activator of interferon induced protein kinase A	PRKRA (PACT)	RIG-I	**MW591865**	8575	557370	(18, 72)
DEAD-box helicase 23	DDX23	MAVS	**MW591869**	9416	334283	(73)
DExH-box helicase 9	DHX9	MAVS	**MW591876**	1660	568043	(74–76)
DEAH-box helicase 15	DHX15	MAVS	**MW591867**	1665	321931	(77–79)
YOD1 deubiquitinase	YOD1 (OTUD2)	MAVS	**MW591873**	55432	550411	(80)
Polo like kinase 1	PLK1	MAVS	**MW591874** ^2^	5347	280649	(81)
OTU deubiquitinase 1	OTUD1	MAVS IRF3	**MW591870**	220213	100537398	(82, 83)
DEAD-box helicase 3	DDX3	MAVS TBK1	LT174679	1654	566947	(24, 75, 84–88)
TNF alpha induced protein 3	TNFAIP3 (A20)	TBK1	LT984694	7128	564497	(89–91)
Elongation factor Tu GTP binding domain containing 2	EFTUD2	TBK1	LT174678	9343	393480	(24, 92)
Protein phosphatase, Mg2+/Mn2+ dependent 1A	PPM1A	TBK1	LT174675	5494	30704	(24, 93–95)
Protein phosphatase, Mg2+/Mn2+ dependent 1B	PPM1B	TBK1	LT174674	5495	100003481	
DEAD-box helicase 19	DDX19	TBK1	**MW591875**	55308	192339	(96)
Optineurin	OPTN	TBK1	**MW591877**	10133	336159	(97–101)
Rapunzel 5	RPZ5	IRF7	**MW591871**	No ortholog	100003142	(24, 102)
Ubiquitin specific peptidase 22	USP22	IRF3	**MW591872**	23326	692275	(103, 104)

^1^ GenBank accession number; ^2^ from Danio rerio; new sequences deposited in the GenBank database are in bold.

RIG-I and MDA5 recognize specific RNA features that are not typically found in most cellular RNAs in the cytoplasm of vertebrate cells (107). RIG-I binds preferentially, but not exclusively, to ssRNAs phosphorylated at the 5’ end, whereas MDA5 recognizes long dsRNAs. This difference in ligand preference results in specificity for the recognition of distinct virus species. In the resting state, CARDs are sequestered, while upon binding of RNA to CTD and helicase domains, CARDs are released by a conformational change of the molecule. Exposed CARDs interact with the CARD of the mitochondrial activator of virus signaling (MAVS) protein (IPS-1, VISA or Cardif) (29–32). MAVS is an integral protein of the mitochondrial outer membrane that associates with the mitochondrial membrane *via* its C-terminal domain and acts as a key determinant of the antiviral signaling cascade. Fish MAVS contains similar domains as those found in mammals, with a N-terminal CARD domain and a C-terminal transmembrane (TM) region, both of which are essential for its antiviral function, as well as a central proline-rich region containing TNF receptor-associated factor (TRAF)-binding motifs (4, 25). The interaction between RLRs and MAVS induces the recruitment of adaptor proteins, such as TRAF3 or TRAF6, and the activation by phosphorylation of serine/threonine-protein kinases, TANK-binding kinase 1 (TBK1) and inhibitor-κB kinases. Consequently, IRF3/IRF7 and nuclear factor kappa-light-chain-enhancer of activated B cells (NF-κB) transcription factors are activated, translocate from the cytosol to the nucleus and induce the expression of IFNs and inflammatory cytokines.

## Regulation of RIG-I-Like Receptors

Under homeostatic conditions, IFNs are expressed at very low and often undetectable levels. Given the critical role of the RIG-I-mediated IFN induction pathway, a tight regulation is essential to maintain the immune homeostatic balance and to ensure proper termination of the antiviral response in order to avoid extensive tissue damage, chronic inflammation, and autoimmune diseases. Moreover, since most RIG-I pathway components are ISGs and that their overexpression leads to constitutive IFN production, it is clear that cells must regulate them not only at the transcriptional level, but also at post-transcriptional and post-translational levels. These distinct regulatory mechanisms act on each sensor and downstream molecule to control antiviral signaling. Regulation at the post-transcriptional level includes alternative pre-mRNA splicing leading to functionally distinct proteins (108), long non-coding RNAs (lncRNAs), and micro RNAs (miRNAs) that both serve as important regulators of RLR signal transduction (5). Some lncRNAs have even been shown to directly bind to RLRs. Post-translational modifications (PTMs) involve the covalent linkage of new functional groups to amino acid residues which in turn fine tune protein properties by regulating protein folding, stability, location, and interaction with other molecules. Several regulatory mechanisms mediated by PTMs have been described (109). Among them, phosphorylation and ubiquitination are the best characterized. Other PTMs such as ISGylation (conjugation with the IFN-inducible ubiquitin-like protein ISG15), SUMOylation, methylation, acetylation and deamidation have also been reported to control the RIG-I pathway. In addition, several RLR-binding proteins have been identified as important modulators of RLRs for RNA binding (acting as co-receptors), oligomerization, ubiquitination or affecting subcellular localization (5). In addition, spatiotemporal dynamics of MAVS in mitochondria, in mitochondrial-associated endoplasmic reticulum membranes (MAMs), and in peroxisomes regulates RLR-mediated signaling (110). Therefore, the integrity of these subcellular compartments together with their own regulation indirectly act on RLR function (111, 112). A few examples of such mechanisms have been described in fish cells, mainly miRNA-mediated regulation and alternative splicing isoforms of RLR components (113, 114).

In order to explore the degree of conservation of these regulatory mechanisms among vertebrates, we cloned and fully sequenced 22 genes of fathead minnow (*Pimephales promelas*) encoding orthologs of human proteins described as important regulators of the RIG-I pathway (**Table 1**). Fathead minnow is a relevant fish species for at least two reasons: 1) EPC cells (*Epithelioma Papulosum Cyprini*; ATCC CRL-2872), the most widely used fish cell line in virology, is derived from this fish species (115), and 2) fathead minnow belongs to the family Cyprinidae together with the zebrafish (*Danio rerio*), an animal model offering great potential for the study of human and fish viral diseases and the development of antiviral drugs (116–118). This list of 22 fish orthologs is far from exhaustive and only represents a small fraction of proteins described as modulating RIG-I-mediated IFN expression (109, 119). Nevertheless, these orthologs are of importance because they correspond to human proteins acting on the RLR pathway *via* three key modes of action: phosphorylation, ubiquitination, and RLR-binding.

## Regulation by Kinases and Phosphatases

Phosphorylation is a reversible PTM of proteins in which serine, threonine or tyrosine residues are modified by a kinase by the addition of a covalently bonded phosphate group (109). Phosphorylation results in a structural conformation change of a protein, often modifying its function to become activated or deactivated. The reverse reaction of phosphorylation is called dephosphorylation, and is catalyzed by phosphatases. Phosphorylation regulates almost all components of the RLR pathway. In resting cells, RIG-I is negatively regulated by phosphorylation by several kinases keeping RIG-I in a non-activated state. When viral RNAs are detected, the CARDs of RIG-I are rapidly dephosphorylated by protein phosphatase 1 (PP1A), thus activating the sensor (70). However, PP1A is also able to dephosphorylate IRF3/7 leading to an inhibition of RIG-I-mediated signal transduction at a downstream level (71). Fish PP1A is highly conserved and share 90% sequence identity at the amino acid (aa) level with its human ortholog. To determine fish PP1A action on RIG-I-mediated IFN expression, we tested its ectopic overexpression in a cell-based luciferase reporter system (**Figure 1B**). As previously published (48), the expression of a constitutively active form of RIG-I (RIG-I Nter; in which the C-terminal repressor domain maintaining the protein in an inactive state is deleted) significantly activates the IFN1 promoter of EPC cells. As a control, the co-expression RIG-I Nter with A20, a negative feedback regulator of the RLR signaling (89), drastically reduced the induction. In contrast, co-expression of PP1A with RIG-I Nter significantly increase IFN1 promoter activation, indicating that fish PP1A share a common function with its mammalian orthologs by enhancing RIG-I activity. MAVS activation is also regulated by phosphorylation (120). Polo-like kinase 1 (PLK1) has been reported to negatively regulate MAVS (81). PLK1 does not directly phosphorylate MAVS but, rather, requires phosphorylation of MAVS for docking and disrupting the MAVS–TRAF3 interaction. Fish PLK1 is well conserved (71% aa sequence identity) and also exerts a negative regulatory role on MAVS (**Figure 1C**). The last example is TBK1. As a critical kinase involved in IFN expression, the activity of TBK1 must be tightly regulated. Because TBK1 activation occurs by trans-autophosphorylation, phosphatases play a critical role in the control of TBK1 activity. Two Ser/Thr protein phosphatases, PPM1A and PPM1B, have been reported to target TBK1 and MAVS for dephosphorylation and to down regulate signaling mediated by cytosolic nucleotide sensing in fish and mammalian species (**Figure 1C**) (24, 93–95).

## Regulation by Ubiquitin Ligases and Deubiquitinases

Ubiquitination is the covalent and reversible addition of ubiquitin to lysine residues on a protein substrate (121). Ubiquitin is itself an 8.5 kDa protein composed of 76 amino acids. Ubiquitination is catalyzed by three distinct classes of enzymes: ubiquitin-activating enzymes (E1), ubiquitin-conjugating enzymes (E2) and ubiquitin ligases (E3), on which lies most of the substrate specificity. Lysine residues can be modified with a single ubiquitin (monoubiquitination) or chains of ubiquitin (polyubiquitination). Different types of ubiquitin chains are thus generated based on the seven lysine residues present on ubiquitin. Among them, K48-linked ubiquitin chains target protein for proteasome degradation while K63-linked ubiquitin chains mediate protein-protein interactions. Ubiquitination is a reversible and dynamic event, since the conjugated ubiquitin chains can be cleaved by a family of ubiquitin-specific proteases, termed deubiquitinases (DUBs). More than 600 and 100 genes encoding putative E3 ligases and DUBs, respectively, have been annotated in the human genome, indicating the ubiquitous importance and specificity of these PTMs in the control of cellular processes. In the RLR pathway, most of the sensors, adaptor proteins, and kinases are ubiquitinated to efficiently activate or repress IFN production.

RIG-I is finely regulated by ubiquitination which is critical for its activation and degradation. Tripartite motif containing 25 (TRIM25) was the first identified enzyme to catalyze the conjugation of K63-linked ubiquitin chains to RIG-I CARDs (64, 65). Ring finger protein 135 (RNF135/RIPLET), another ubiquitin ligase, is also involved in K63-linked polyubiquitination at multiple sites in CARDs and CTD leading to the activation of RIG-I (58–60). Whether RNF135 promotes TRIM25 binding on RIG-I in a sequential ubiquitination process or RNF135 by itself, without involvement of TRIM25, is essential for RIG-I activation is still unclear (61, 62). However, TRIM25 is also capable of promoting K48-linked ubiquitination and degradation of MAVS. The proteasomal degradation of MAVS is required to release the signaling complex into the cytosol, allowing IRF3 phosphorylation by TBK1 (23). Zebrafish orthologs of TRIM25 and RIPLET have also been reported as positive regulators of RIG-I (63, 66). **Figure 1B** shows that fish RNF135 has an enhancing effect on the activity of RIG-I CARDs, whereas TRIM25 has no effect. Nevertheless, TRIM25 co-expression with full-length RIG-I is required to protect EPC cells against a viral infection and to inhibit viral production (**Figures 1D, E**), highlighting that fish RNF135 and TRIM25 are both positive regulators of the RLR pathway.

Several DUBs of ovarian tumor proteases (OTUs) and ubiquitin-specific proteases (USPs) families, have been described as important regulators of RLR pathway. Among them, mammalian and fish A20 has been shown to be a strong inhibitor of the RLR signaling (**Figures 1C, D**) (89–91). In addition, the function of OTUB1, OTUD1, YOD1, and USP22 fish orthologs was investigated (**Table 1**). They all have significant inhibitory effects on signal transduction by RIG-I CARDs (**Figure 1C**). Fish OTUD1 has the strongest effect, likely by mediating the targeted degradation of the MAVS/TRAF3/TRAF6 signalosome as well as by reducing the DNA binding capacity of IRF3, as described in mammals (82, 83). YOD1, which acts at a later step along the pathway to abrogate the formation of prion-like aggregates of MAVS (80), has a limited effect on IFN promotor induction at an early time point post-stimulation. In contrast, mammalian OTUB1 and USP22 were reported with opposite regulatory effects on the RLR pathway (67–69, 103, 104). The inhibitory effect observed after ectopic expression of the fish orthologs may be a result of the inherent bias associated with the overexpression of enzymatically-active protein, mislocalization and inadequate cell type and does not allow to distinguish the opposite functions previously described in mammals.

## Regulation by RLR-Binding Proteins

The RLR pathway is regulated by multiple host factors. Protein activators of PKR (PACT, also known as protein activator of interferon induced protein kinase A) binds to RIG-I CTD and enhances RIG-I signaling in part by stimulating RIG-I ATPase and helicase activities (72). Moreover, recent studies have indicated that the PACT-LGP2 interaction was necessary to regulate the responses mediated by RIG-I and MDA5 (18, 122). As for mammals, the role of fish LGP2 in RLR signaling is unclear. It appears that depending on the nature of the splicing isoform, LGP2 can have a negative or a positive effect on the RIG-I pathway (27, 49, 53–55). The *dhx58* cDNA amplified from EPC cells encodes LGP2 protein which exerts a strong inhibition on signaling mediated by RIG-I CARDs (**Figure 1B**). Moreover, a similar inhibition is observed during expression of RIG-I CARDs together with PACT (**Figure 1B**). This is in contrast with PACT’s enhancing function observed in mammals. However, fish PACT only shares 44% aa sequence identity with human PACT. Another dsRNA-binding protein, TAR-RNA-binding protein (TRBP), which shares 39% protein sequence identity with PACT with a similar structure, has recently been reported as an inhibitor of RIG-I signaling (123). Because fish PACT still retains some degree of relatedness to both human proteins, PACT and TRBP, it cannot be excluded that PACT acts as a negative regulator of RIG-I in fish.

The involvement of multiple RNA helicases in RLR signaling has been demonstrated, as recently reviewed by Taschuk and Cherry (124). For instance, DDX6, DHX9, DDX3, and DHX15 can function as co-sensors of RIG-I or as RLR-independent sensors of nucleic acids through interaction with MAVS (56, 74, 77–79, 84–86). DHX9, DHX15, and DDX23 have been recently described as cytoplasmic viral RNA sensors in the lancelet (amphioxus) (73). However, limitations or contradictions have been reported concerning their role in IFN and ISGs production. DDX6 is also described as a suppressor of ISGs (57). DHX9 is an important viral dsRNA sensor only in myeloid dendritic cells (74). DHX15 contributes to the activation of NF-κB but not IRF3 in response to RNA virus infection (78). DDX3, for which multiple roles as a pro- or antiviral factor were identified (84), has recently been described as an inhibitor of IFN production during arenavirus infection (87). Fish orthologs are highly conserved and share at least 69% aa sequence identity with human proteins. Fish DDX3 and DHX9 bind dsRNA (75) and DHX9 is a potential sensor for DNA virus infection *in vivo* (76). Fish DDX3 is a binding partner for the nonvirion (NV) proteins of two fish novirhabdoviruses, suggesting that DDX3 plays an important role in either enhancing innate immunity or promoting virus replication (24). Moreover, the overexpression of fish DDX3 alone seems to induce the IFN promoter (88). In our cell-based reporter system, a negative effect on RIG-I CARDs-mediated signaling was observed for DDX6, DHX9, DDX3, DHX15, and DDX23 (**Figures 1B, C**), probably through a competition for MAVS adaptor or another mechanism yet to be further investigated. In any case, these RNA helicases are potentially involved in the innate immune system of vertebrates. Finally, another RNA helicase, DDX19, has been shown as a negative regulator of IFN production (96). Mechanistically, DDX19 does not sense viral RNA but inhibits the phosphorylation of IRF3 by TBK1. DDX19 is highly conserved between fish and mammals (sharing 86% aa sequence identity) and share the same inhibitory effect on the RLR pathway (**Figure 1C**).

The optineurin (OPTN) is another regulator of the RLR pathway but its action is controversial. Although OPTN was initially reported to negatively regulate IFN induction (97), other studies indicated that OPTN was necessary for optimal TBK1 and IRF3 activation (98, 101). However, recent studies pointed out a crucial role for OPTN in dampening the IFN response (99, 125). Moreover, chicken OPTN has been reported as an inhibitor of MDA5-mediated IFN production (100). As shown in the **Figure 1C**, fish OPTN has also an important inhibitory effect on RIG-I-mediated induction of the IFN promoter.

The function of the NV proteins of two novirhabdoviruses in the inhibition of the host immune response has been described using an interactome proteomics approach (24). Among the cellular partners of NV, PPM1B was shown to be specifically recruited to terminate RIG-I-mediated IFN induction. In addition to DDX3, two other proteins were identified to be likely involved in the RLR pathway: the elongation factor Tu GTP binding domain containing 2 (EFTUD2) and the rapunzel 5 protein (RPZ5). EFTUD2 was discovered to restrict infection by hepatitis C virus (HCV) through IFN-independent stimulation of the innate immune response (92). EFTUD2 upregulates RIG-I expression by pre-mRNA splicing. Fish EFTUD2 is highly conserved with its mammalian counterpart (89% aa sequence identity) but its overexpression does not protect fish cells against rhabdovirus infection in contrast to its human ortholog that protects human cells against HCV (data not shown). Surprisingly, overexpression of EFTUD2 has a negative effect on RIG-I-mediated IFN expression in fish cells (**Figure 1C**), a finding that requires further investigation. Unlike most of the factors described above, RPZ5 has no mammalian or bird orthologs. Zebrafish RPZ5 has recently been implicated in blocking RLR-mediated IFN induction by mediating the degradation of phosphorylated IRF7 (102). In **Figure 1C**, we confirm the inhibitory effect of fish RPZ5 on the RIG-I pathway and its uniqueness among teleost fish.

## Conclusions and Perspectives

The IFN system is remarkably well conserved in vertebrates and it is remarkable that teleost fish possess most post-transcriptional and post-translational regulatory mechanisms of the RLR signaling pathway as described in mammals. Thus, these multi-level regulatory mechanisms were selected very early on and maintained throughout the evolution of vertebrates indicating their crucial role in the control of immune homeostasis for these organisms. Although numerous regulators have been reported in mammals, underlying the complexity and the relative redundancy of these mechanisms, their distinctive roles and functional differences depending on the cell type considered (*e.g*. immune versus epithelial cells), their own regulation, and their sequential chronology required to orchestrate the RLR signaling remain elusive, and in some cases, opposite functions have been reported for a same effector. In teleost fish, characterization of the components of the RLR pathway and factors involved in its fine tuning has begun but the overall picture is still poorly understood and is mainly modeled on the knowledge acquired from studies based on mammalian systems. The experimental approaches to study the innate immune system in fish has long been based on the overexpression in cell lines of identified genes with the known benefits and limitations of a such screening method. However, with the adaptation of the CRISPR/Cas9 genome editing for fish cells (126, 127), gene knock out studies will be greatly improved compared to the low efficiency and biases observed with RNA silencing (128). Moreover, the *in vivo* relevance of these factors in antiviral immunity still needs to be addressed since their description was exclusively done *in vitro* in non-immune cells. Since many decades, zebrafish is an important animal model in biomedical research due to multiple advantages including low maintenance cost, high fecundity, short generation time, small size, optical transparency of embryos, and a relatively high degree of conservation with human genes (**Table 1**) (129, 130). Together with the large available collection of transgenic lines and the relative ease to silence or overexpress specific genes, these advantages make zebrafish a model of choice for studying the spatio-temporal regulatory mechanisms of the RLR pathway. An improved understanding of the precise mechanisms of regulation in different viral and animal species and cell types will enable the development of novel therapeutic strategies against infectious diseases, immunological disorders, and cancer.

## Data Availability Statement

The datasets presented in this study can be found in online repositories. The names of the repository/repositories and accession number(s) can be found below: https://www.ncbi.nlm.nih.gov/genbank/, MN781134 MN781135 MW591879 MW591868 MW591864 MW591878 MW591866 MW591865 MW591869 MW591876 MW591867 MW591873 MW591874 MW591870 MW591875 MW591877 MW591871 MW591872.

## Author Contributions

All authors listed have made a substantial, direct and intellectual contribution to the work, and approved it for publication.

## Funding

This work was supported by the institut de recherche pour l’agriculture, l’alimentation et l’environnement (INRAE). RJ is the recipient of a Ph.D. fellowship from the Doctoral School ABIES, AgroParisTech.

## Conflict of Interest

The authors declare that the research was conducted in the absence of any commercial or financial relationships that could be construed as a potential conflict of interest.
